# An improved scheme on decoy-state method for measurement-device-independent quantum key distribution

**DOI:** 10.1038/srep15130

**Published:** 2015-10-14

**Authors:** Dong Wang, Mo Li, Guang-Can Guo, Qin Wang

**Affiliations:** 1 Institute of Signal Processing Transmission, Nanjing University of Posts and Telecommunications, Nanjing 210003, China; 2 Key Lab of Broadband Wireless Communication and Sensor Network Technology, Nanjing University of Posts and Telecommunications, Ministry of Education, Nanjing 210003, China; 3 Key Laboratory of Quantum Information, University of Science and Technology of China, Hefei 230026, China; 4 Synergetic Innovation Center of Quantum Information & Quantum Physics, University of Science and Technology of China, Hefei, Anhui 230026, China

## Abstract

Quantum key distribution involving decoy-states is a significant application of quantum information. By using three-intensity decoy-states of single-photon-added coherent sources, we propose a practically realizable scheme on quantum key distribution which approaches very closely the ideal asymptotic case of an infinite number of decoy-states. We make a comparative study between this scheme and two other existing ones, i.e., two-intensity decoy-states with single-photon-added coherent sources, and three-intensity decoy-states with weak coherent sources. Through numerical analysis, we demonstrate the advantages of our scheme in secure transmission distance and the final key generation rate.

Quantum key distribution (QKD) entails two legitimate parties, Alice and Bob, to distribute secure keys in the presence of an eavesdropper, Eve^1^. The security of QKD has been established theoretically by virtue of the principle of quantum mechanics^2^,^3^,^4^. However, the security claims are based on theoretical and idealized assumptions, such as some convenient models on the photon sources or the detectors, which are not necessarily met by experimental implementations. In experiment, one usually adopts the weak coherent state (WCS) generated from attenuated lasers to replace the ideal single-photon source, which is unavailable at present. Nevertheless, there are non-negligible multi-photon components in WCS, which can be exploited by Eve via the photon-number-splitting (PNS) attack^5^,^6^,^7^.

To combat the PNS attack, the powerful decoy-state method is proposed^8^,^9^,^10^,^11^,^12^,^13^,^14^,^15^,^16^,^17^. Then more work about the decoy-state method with an arbitrary number of intensities and related security analysis for finite key length have been discussed^18^,^19^,^20^. The decoy-state method can be further combined with the newly proposed measurement-device-independent quantum key distribution (MDI-QKD) to fight all other potential detector side-channel attacks^21^,^22^,^23^,^24^,^25^,^26^,^27^. Through the decoy-state method, one can estimate the lower bound of the counting rate and the upper bound of the quantum-bit error-rate (QBER) caused by two-single-photon pulses, and thus obtain a lower bound for the secure key rate. In order to get more precise estimations, one can use better light sources with negligible vacuum component and multi-photon probabilities^15^,^16^, or use more intensities of decoy-states^11^,^25^. Large number of intensities of decoy-states will cause experimental difficulties and larger statistical fluctuations. In this report, by using single-photon-added coherent sources (SPACS)^28^,^29^, we propose a scheme involving only three intensities of decoy-states which nevertheless can approach very closely the asymptotic case involving infinite number of intensities.

SPACS has a relatively high probability of single-photon and no vacuum component. In principle, the state 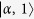 of SPACS can be generated by the elementary one-photon excitation on a coherent state^28^,^29^,^30^, and is theoretically described by applying the photon creation operator 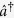 to a coherent state 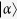:


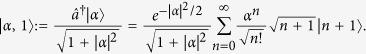


It is clear that there is no vacuum term contribution in the state of SPACS. The probability of finding *n* photons is


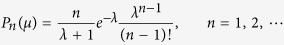


where 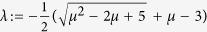 and *μ* is the mean photon number. SPACS has been experimentally created with high efficiency and fidelity^29^,^30^,^31^,^32^,^33^,^34^. In particular, Zavatta *et al*. prepared the SPACS by a conditional technique through parametric down-conversion process^30^,^34^, where a piece of LBO crystal is pumped with a Ti:sapphire laser working at 393 nm, and the generated SPACS is working at 786 nm, the overall efficiency obtained is 60%, and the corresponding state fidelity is up to 99.5%. In general, almost all the conditions required for QKDs had been matched except for the signal wavelength. Nevertheless, we find no in-principle difficulty in generating the SPACS at telecommunication wavelength since what we need is only to change the phase-match conditions inside nonlinear crystals, e.g., replacing LBO with PPKTP. Therefore, it is feasible to apply SPACS to QKD under present technology.

In this report, we apply SPACS to MDI-QKD by using three-intensity and combining the method proposed by Zhou *et al*.^25^. Due to the absence of vacuum component in SPACS, we need not take the contribution of vacuum pulses into account as in other schemes. Using only three non-zero intensities (two decoy-states and one signal state) of SPACS, we can get precise estimation of the counting rate and the quantum bit error rate (QBER) caused by single-photon pulses, which leads to significantly improved final key generation rate and secure transmission distance.

For our scheme, we will need the following results. First, when 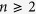 and 1 ≤ *μ*_*x*_ < *μ*_*y*_, the photon number distribution in a state of SPACS has the following property


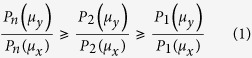


which follows from


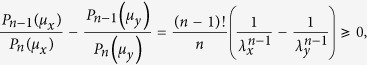


where 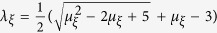, 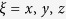. The last inequality is ensured by *λ*_*x*_ < *λ*_*y*_ since 1 ≤ *μ*_*x*_ < *μ*_*y*_. Next, when *i* ≤ *j* ≤ *k* and *μ*_*x*_ ≤ *μ*_*y*_ ≤ *μ*_*z*_, it holds that


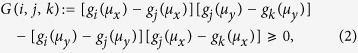


where 
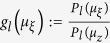
, 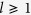, 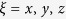. To prove this, note that


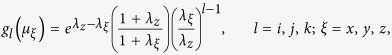


and *G*(*i*, *j*, *k*) can be rewritten as


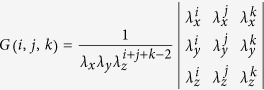


which is positive due to the property of generalized Vandermonde determinant and the conditions *i* ≤ *j* ≤ *k, λ*_*x*_ < *λ*_*y*_ < *λ*_*z*_.

## Improved 3-intensity decoy-state method for MDI-QKD

**Figure 1:**
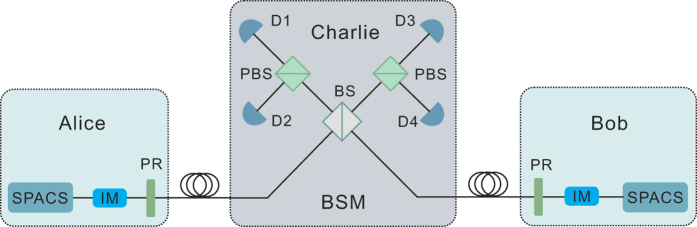
A schematic setup of MDI-QKD using the SPACSs. Alice and Bob randomly prepare SPACSs in a BB84 polarization state with a polarization rotator (PR). Intensity modulator (IM) is used to generate decoy-states. Charlie performs a partial BSM when the signal pulses from Alice and Bob arrive at a 50:50 beam splitter (BS). Four single-photon detectors (D1–D4) are employed to detect the results.

In MDI-QKD, Alice and Bob simultaneously send signals to an untrusted third party (UTP, possibly controlled by an eavesdropper Eve). The UTP performs a partial BSM and announces whether the measurement result is successful. According to the UTP’s announcement, those successful events will be post-selected and further processed for the final key generation by Alice and Bob. A schematic setup of our three-intensity decoy-state MDI-QKD with SPACS is shown in Fig. 1. Alice and Bob need to randomly prepare the signals with intensities *α*, *β*, respectively, where *α*, *β* ∈ {*μ*_*x*_, *μ*_*y*_, *μ*_*z*_}. Here *μ*_*x*_ and *μ*_*y*_ are the intensities of the two decoy-states, while *μ*_*z*_ is the intensity of the signal state, *μ*_*x*_ < *μ*_*y*_ < *μ*_*z*_. When Alice and Bob send signals with intensities *α* and *β*, respectively, the gain and QBER are given by


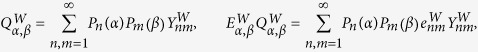


respectively. Here *W* represents the *Z*- or *X*-basis, and *n*,  *m* denote the number of photons sent by Alice and Bob, respectively. 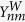 denotes the yield, and 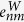 denotes the error rate, when Alice sends an *n*-photon pulse and Bob sends an *m*-photon pulse to the UTP. The decoy-states and signal-state are prepared in different bases. Hereafter we shall omit the superscript *W* without causing any confusion.

As demonstrated in ref. 25, as long as inequalities (1) and (2) are satisfied, we can get the lower bound of *Y*_11_ by using the lowest two intensities (*μ*_*x*_ and *μ*_*y*_) for Alice and Bob such that


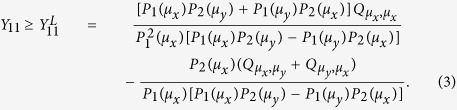


Moreover, we can get an upper bound of *e*_11_ by inequalities (1) and (2) as^25^


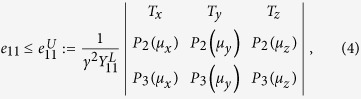


where *γ* = *P*_1_(*μ*_*z*_)*P*_2_(*μ*_*z*_)*P*_3_(*μ*_*z*_)*G*(1, 2, 3), and for *ξ* ∈ (*x*, *y*, *z*),


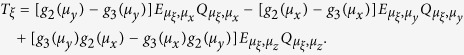


In our protocol, the *Z*-basis is used as the key generation basis, and the *X*-basis is for error testing only. Then by inequalities (3) and (4), one can obtain the lower bound of the successful single-photon yield 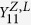 in the *Z*-basis and the upper bound of the single-photon error rate 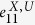 in the *X*-basis. The final secure key rate can be calculated with the observed total gains and error rates as


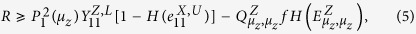


with *f* being the error correction efficiency and *H*(*p*) := −*p* log_2_(*p*) − (1 − *p*)log_2_(1 − *p*) is the binary Shannon entropy function.

## Numerical Simulation

**Figure 2:**
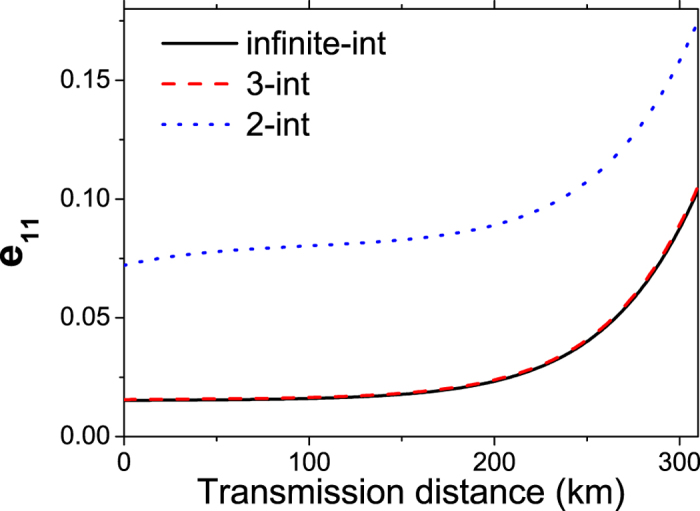
Comparison of the estimated values of *e*_11_ for MDI-QKD with SPACS by using different number of decoy states. The dashed curve represents the result of our three-intensity decoy-state method, the solid curve represents the result of using an infinite number of decoy-states, and the dotted curve corresponds to the result of two-intensity decoy-states method.

**Figure 3:**
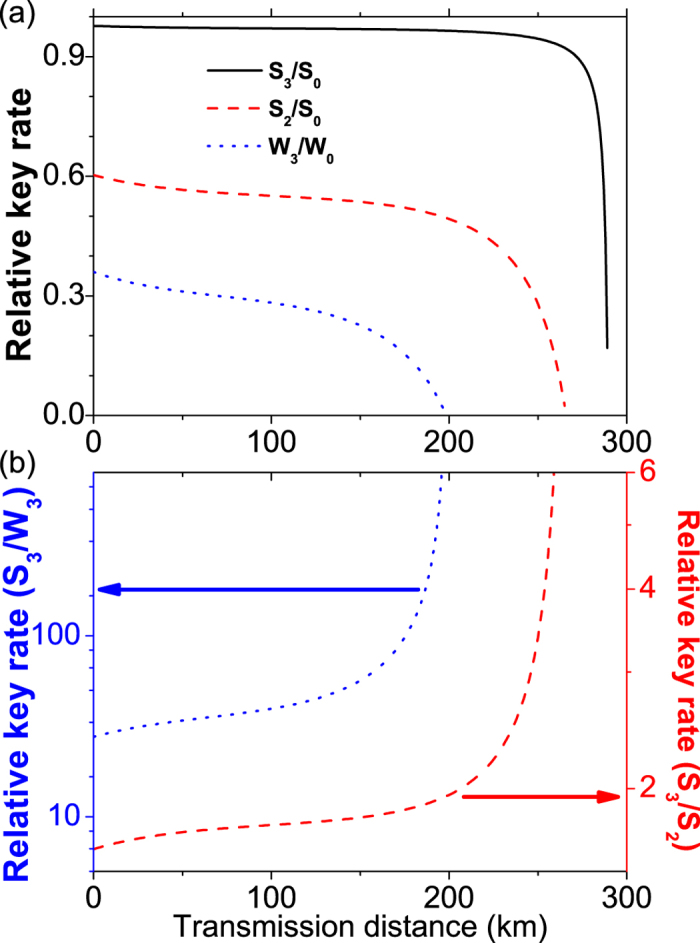
The relative key generation rates of different decoy-state MDI-QKD protocols, either with SPACS or WCS. *S*_3_, *S*_2_ or *S*_0_ represents the key generation rate for MDI-QKD involving three-intensity, two-intensity or infinite decoy-state, with SPACS. *W*_3_ and *W*_0_ are the corresponding key generation rates with WCS. (**a**) Comparison of the normalized key generation rate for different methods, i.e., two- or three-intensity decoy-state SPACS, or the three-intensity decoy-state WCS. (**b**) The ratio of the key generation rates between our scheme and the conventional two-intensity decoy-state with SPACS or the three-intensity decoy-state with WCS.

With inequalities (3–5) we can perform corresponding numerical simulation for our three-intensity MDI-QKD with SPACS. We further compare our scheme with the two-intensity MDI-QKD involving SPACS^35^ and the conventional three-intensity MDI-QKD involving WCS^23^. For the total gains and error rates, which can be directly measured from the experiment, we use the channel model and method as in^27^ to estimate these values. The relevant parameters are listed in Table 1 21. During the simulation, for the two-intensity or our three-intensity decoy-states with SPACS, we set reasonable intensities with *μ*_*x*_ = 1.05, *μ*_*y*_ = 1.06 for the decoy-states, and *μ*_*z*_ = 1.10 for the signal-state. For the three-intensity decoy-states with WCS, we set *μ*_*x*_ = 0, *μ*_*y*_ = 0.1 for decoy-states, and optimize the intensity for the signal-state (*μ*_*z*_) in each instance. Corresponding simulation results are shown in Figs 2 and 3.

In Fig. 2, we compare the estimation value of *e*_11_ between our three-intensity decoy-state method and the conventional two-intensity decoy-state method when both using SPACS. Obviously, by using our three-intensity decoy-state method, we can get significant improvement in the estimation of *e*_11_ over the conventional two-intensity decoy-state method. Moreover, our method approaches very closely the ideal value by using an infinite number of intensities of decoy-states.

In Fig. 3(a), we give the comparison of the key generation rates by using different methods, i.e., our three-intensity decoy-state with SPACS, the conventional two-intensity decoy-state with SPACS, and the three-intensity decoy-state with WCS. In each case the key generation rate has been normalized by the corresponding value of using an infinite number of intensities of decoy-states. We find from Fig. 3(a) that our scheme performs much better than the other two methods: Longer secure transmission distance and much higher key generation rate. In Fig. 3(b), the ratio of the key generation rate between our scheme and the other two methods have also been displayed. It can be seen that our scheme shows excellent behavior even at rather long distance (>200 km). It exhibits tens of times or even hundreds of times of enhancement in the key generation rate than the three-intensity decoy-state method with WCS at long distances (>150 km), see the left axis of Fig. 3(b). When compared with the conventional two-intensity decoy-state method with SPACS, our scheme obtains more than double enhancement in the key generation rate at very long distances (>200 km), see the right axis of Fig. 3(b).

## Conclusion

We have introduced an improved scheme on MDI-QKD involving three-intensity decoy-state with SPACS, and have compared its performance with two existing methods. Through numerical simulation, we have demonstrated that our scheme shows excellent behavior in both the secure transmission distance and the final key generation rate. For example, when compared with the conventional two-intensity MDI-QKD with SPACS, the key generation rate is enhanced by several times. Compared with the three-intensity MDI-QKD with WCS, our scheme not only presents almost one hundred kilometers increasing in the secure transmission distance, but also shows tens of times enhancement in the final key generation rate. We emphasize that our scheme depends on SPACS which can be generated with current technology, although its present setup is relatively bulky and has higher technical requirements compared with the WCS system. We can expect that with the development of technology, the emergence of miniaturization and maturing of SPACS system will cause it to replace other sources and launches a wide implementation in quantum key distributions in the near future.

## Additional Information

**How to cite this article**: Wang, D. *et al*. An improved scheme on decoy-state method of measurement-device-independent quantum key distribution. *Sci. Rep*. **5**, 15130; doi: 10.1038/srep15130 (2015).

## Figures and Tables

**Table 1:** Parameters values for simulations.

*η*_*d*_	*Y*_0_	*e*_*d*_	*e*_0_	*α*	*f*
14.5%	3.0 × 10^−6^	1.5%	0.5	0.2 dB/km	1.16

*η*_*d*_ and *Y*_0_ are the transmittance and dark count rate; *e*_*d*_ is the probability that the survived photon hits a wrong detector, which is independent of the transmission distance, and *e*_0_ is the error rate of dark count; *α* is the transmission fiber loss constant; *f* is the error correction efficiency. The UTP is located midway between Alice and Bob, and all detectors are identical.
